# Downregulation of TRAIL-Receptor 1 Increases TGFβ Type II Receptor Expression and TGFβ Signalling Via MicroRNA-370-3p in Pancreatic Cancer Cells

**DOI:** 10.3390/cancers10110399

**Published:** 2018-10-25

**Authors:** David I. Radke, Qi Ling, Robert Häsler, Gökhan Alp, Hendrik Ungefroren, Anna Trauzold

**Affiliations:** 1 Institute for Experimental Cancer Research, University of Kiel, D-24105 Kiel, Germany; david.radke@gmx.de (D.I.R.); lingqi@zju.edu.cn (Q.L.); alp77@gmx.de (G.A.); 2 Department of Surgery, Collaborative Innovation Center for Diagnosis and Treatment of Infectious Diseases, the First Affiliated Hospital, School of Medicine, Zhejiang University, Hangzhou 31000, China; 3 Institute of Clinical Molecular Biology, University of Kiel, D-24105 Kiel, Germany; r.haesler@mucosa.de; 4 Clinic for General Surgery, Visceral, Thoracic, Transplantation and Pediatric Surgery, University Hospital Schleswig-Holstein, Campus Kiel, D-24105 Kiel, Germany; hendrik.ungefroren@uksh.de; 5 First Department of Medicine, University Hospital Schleswig-Holstein, Campus Lübeck, D-23538 Lübeck, Germany

**Keywords:** TRAIL, TRAIL-receptor 1, TGFβ, TGFβ receptor II, microRNA, signalling, pancreatic ductal adenocarcinoma

## Abstract

The accumulation of perturbations in signalling pathways resulting in an apoptosis-insensitive phenotype is largely responsible for the desperate prognosis of patients with pancreatic ductal adenocarcinoma (PDAC). Accumulating evidence suggests that the death receptors TRAIL-R1 and TRAIL-R2 play important roles in PDAC biology by acting as either tumour suppressors through induction of cell death or tumour promoters through induction of pro-inflammatory signalling, invasion and metastasis. TRAIL-R2 can also associate with nuclear proteins and alter the maturation of micro RNAs (miRs). By genome-wide miR profiling and quantitative PCR analyses we now demonstrate that knockdown of TRAIL-R1 in PDAC cells decreased the level of mature miR-370 and led to an increased abundance of the type II receptor for transforming growth factor β (TGFβ). Transfection of cells with an artificial miR-370-3p decreased the levels of TGFβ-RII. We further show that transient expression of the miR-370 mimic decreased TGFβ1-induced expression of *SERPINE1* encoding plasminogen activator-inhibitor 1 and partially relieved TGFβ1-induced growth inhibition. Moreover, stable TRAIL-R1 knockdown in Colo357 cells increased TGFβ1-induced *SERPINE1* expression and this effect was partially reversed by transient expression of the miR-370 mimic. Finally, after transient knockdown of TRAIL-R1 in Panc1 cells there was a tendency towards enhanced activation of Smad2 and JNK1/2 signalling by exogenous TGFβ1. Taken together, our study reveals that TRAIL-R1 through regulation of miR-370 can decrease the sensitivity of PDAC cells to TGFβ and therefore represents a potential tumour suppressor in late-stage PDAC.

## 1. Introduction

The tumour necrosis factor-related apoptosis-inducing ligand (TRAIL) is a member of the tumour necrosis factor (TNF)-family of ligands [1]. Once bound to its receptors, signalling cascades are initiated leading to apoptosis on the one hand and inflammation, proliferation, or migration on the other hand [2]. TRAIL can bind to five different receptors. The membrane-bound receptors TRAIL-receptor 1 (TRAIL-R1, DR4) [3], -2 (TRAIL-R2, DR5, TRICK2, Killer) [4,5], -3 (TRAIL-R3, DcR1) [6], -4 (TRAIL-R4, TRID, DcR2) [7,8] and the soluble receptor osteoprotegerin (OPG) [9,10]. Since TRAIL preferentially kills tumour cells while sparing normal healthy cells, TRAIL and agonistic anti-TRAIL-R1 and TRAIL-R2 antibodies were developed for treatment of different malignancies [11,12,13]. However, soon thereafter it has been recognized that many tumour cells are resistant to TRAIL-induced apoptosis, the fact explaining the disappointing results from clinical trials [13]. In addition to initiating programmed cell death, TRAIL is also able to promote tumour progression by enforcing inflammation as well as invasion and proliferation of cells [14,15,16,17,18]. These pathways are activated preferentially in cells that are resistant against TRAIL-induced apoptosis.

In addition to the aforementioned functions, in cancer cells TRAIL receptors were also found to be localized in the cytoplasm and in the nucleus [19]. Nuclear localization of TRAIL receptors was demonstrated in pancreatic ductal adenocarcinoma (PDAC), colorectal cancer, mammary carcinoma, hepatocellular carcinoma and melanoma [20,21,22,23]. Recently, it has been shown that nuclear TRAIL-R2 interacts with components of the microprocessor complex, thereby inhibiting the maturation of the microRNA (miR) let-7. This resulted in an increased proliferation rate and enhanced invasion and migration in vitro and in reduced pancreatic tumour growth and breast cancer metastasis into the bone as demonstrated by use of a mouse xenotransplantation model [20,24].

MiRs are small RNA molecules involved in the posttranscriptional regulation of genes, by degrading mRNAs or inhibiting translation [25]. It is assumed that there are 500–1500 miRs in the human genome and that 20–30% of all human genes can be regulated by miRs [26]. Since one particular miR is able to target many different mRNAs, changes in its expression levels may result in a complex network of posttranscriptional gene regulation [27]. Genes encoding miRs are transcribed by RNA polymerase II. Still in the nucleus the so called pri-miR is processed by the microprocessor complex into the precursor (pre-) miR which is 60 to 70 nucleotides long and has a hairpin structure [28]. The main components of the microprocessor complex are Drosha (an RNase III) and DiGeorge syndrome critical region 8 (DGCR8). Several additional proteins associate with the complex and can regulate the maturation of the miR [29]. Once exported to the cytoplasm the pre-miR is cleaved by another RNase III (Dicer) into the mature double-stranded miR [30]. After degradation of one strand, the remaining one is incorporated into the RNA-induced silencing complex (RISC) which is the executive part for posttranscriptional gene regulation [31]. Certain expression patterns of miRs are associated with proliferation, apoptosis or tumour development [32]. In tumour cells the expression of miRs is often altered and miRs have the capacity to act as either an oncogene or a tumour suppressor [33]. Being located in a tumour-associated region on chromosome 14, miR-370 has been linked to tumour pathogenesis [34,35]. Particularly, the miR-370-3p form was described as a tumour suppressor miR due to its low expression in cancers [36,37]. In contrast, other studies report that overexpression of miR-370-3p enhanced tumour progression [38,39].

With a median survival of less than one year after diagnosis, PDAC ranks fourth among tumour-associated death rates [40]. The poor prognosis is due to the fact that most patients already have acquired metastases when diagnosed with this tumour and that the tumour cells have become apoptosis-resistant and refractory to standard chemotherapy [41]. Interestingly, the signalling pathway of transforming growth factor β (TGFβ) is often inactivated in the tumour cells and the genes for the TGFβ type I receptor (TGFβ-RI/ALK5), *TGFBR1* and the type II receptor (TGFβ-RII), *TGFBR2*, are selective targets of genetic inactivation in pancreatic cancers [42]. In addition, the mRNAs encoding TGFβ-RII and TGFβ-RI/ALK5 are directly targeted by several miRs. For TGFβ-RII these include the miR-302/367 cluster, miR-372, miR-520/373, miR-17-92 cluster, miR-15, miR-16 reviewed in Reference [43] and miR-370-3p [44]. Since these miRs are capable of inhibiting TGFβ receptor expression and the receptor levels correlate with TGFβ responsiveness, these miRs control the threshold for signalling initiation in response to TGFβ [43].

Here, we demonstrate that knockdown of TRAIL-R1 in PDAC cells results in decreased amounts of mature miR-370-3p associated with higher expression of TGFβ-RII and enhanced TGF-β target gene expression and growth inhibition.

## 2. Results

### 2.1. TRAIL-R1 Regulates the Expression of miR-370

**Figure 1:**
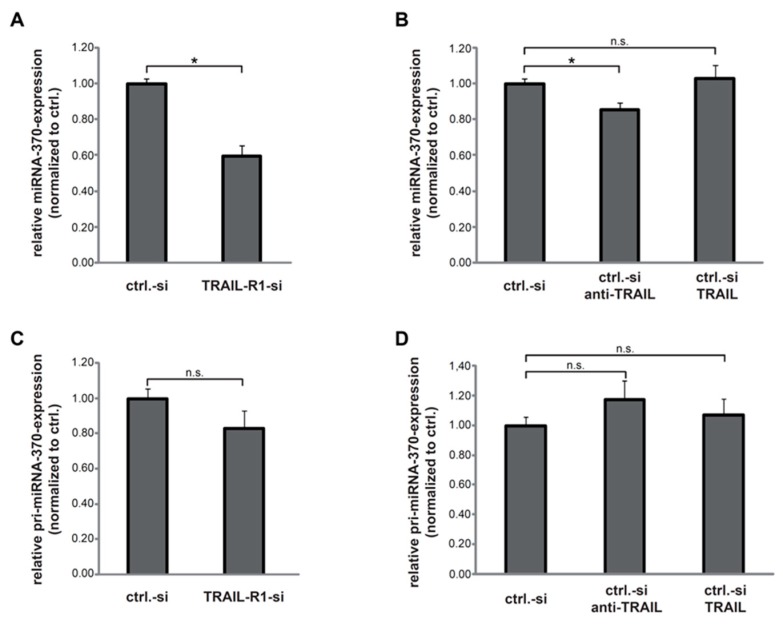
Downregulation of tumour necrosis factor-related apoptosis-inducing ligand-receptor 1 (TRAIL-R1) decreases levels of mature miR-370-3p but not of pri-miR-370. (**A**) Quantitative PCR analyses detecting changes in the levels of mature miR-370-3p in Panc1 cells transfected for 40 h with a control-siRNA (ctrl.-si) or siRNA against TRAIL-R1 (TRAIL-R1-si). (**B**) Panc1 cells transfected with ctrl.-si were stimulated with anti-TRAIL (10 µg/mL), TRAIL (10 ng/mL) or left untreated. Levels of mature miR-370-3p were quantified by qPCR. (**C**,**D**) QPCR analyses of pri-miR-370 levels in Panc1 cells transiently transfected with ctrl.-si or TRAIL-R1-si (**C**) or in ctrl.-si transfected cells with and without treatment with TRAIL (10 ng/mL) or anti-TRAIL (1 µg/mL) (**D**). Shown are the mean ± SD of five biological replicates (*n* = 5), with each one analysed in technical duplicates. The asterisks (*) indicate significance (*p* < 0.05); n.s.: not significant.

To analyse the possible impact of TRAIL-R1 on the expression of miRNAs, we performed genome-wide miR-profiling in Panc1 cells with and without knockdown of TRAIL-R1. Interestingly, miR-370 was among the most strongly downregulated miRs in response to TRAIL-R1 depletion (Figure S1). To verify the array data, we again transfected the Panc1 cells with siRNA against TRAIL-R1 and determined the levels of the mature miR-370-3p by quantitative real-time PCR (qPCR) analysis. As shown in Figure 1A, downregulation of TRAIL-R1 but not TRAIL-R2 (Figure S2), resulted in significantly reduced levels of miR-370-3p. In an attempt to elucidate the underlying mechanism, we first asked whether TRAIL impacts miR-370 expression. Therefore, control siRNA-transfected Panc1 cells were treated with either recombinant TRAIL or a neutralizing antibody against TRAIL (anti-TRAIL). The abundance of miR-370-3p was not affected by treatment with TRAIL (Figure 1B) as no significant changes in expression of mature miR-370 was observed. However, antibody-mediated deprivation of endogenous TRAIL reduced miR-370 levels significantly albeit slightly. These results suggest that mature miR-370-3p is positively regulated by TRAIL-R1 in a manner independent of rec. human TRAIL ligand.

Next, we addressed the question whether TRAIL-R1 regulates miR-370-3p expression at the transcriptional level. For this purpose, we compared the levels of pri-miR-370 in cells with and without knockdown of TRAIL-R1 using qPCR. Although the levels of pri-miR-370 appeared reduced, differences missed statistical significance (Figure 1C). Likewise, neither treatment with anti-TRAIL nor with recombinant TRAIL affected the abundance of pri-miR-370 relative to control siRNA (Figure 1D). These results suggest that neither TRAIL-R1 nor TRAIL (in its exogenous or endogenous form) affects miR-370-3p expression at the transcriptional level.

### 2.2. MiR-370-3p Negatively Controls TGFβ-RII in PDAC Cells

**Figure 2:**
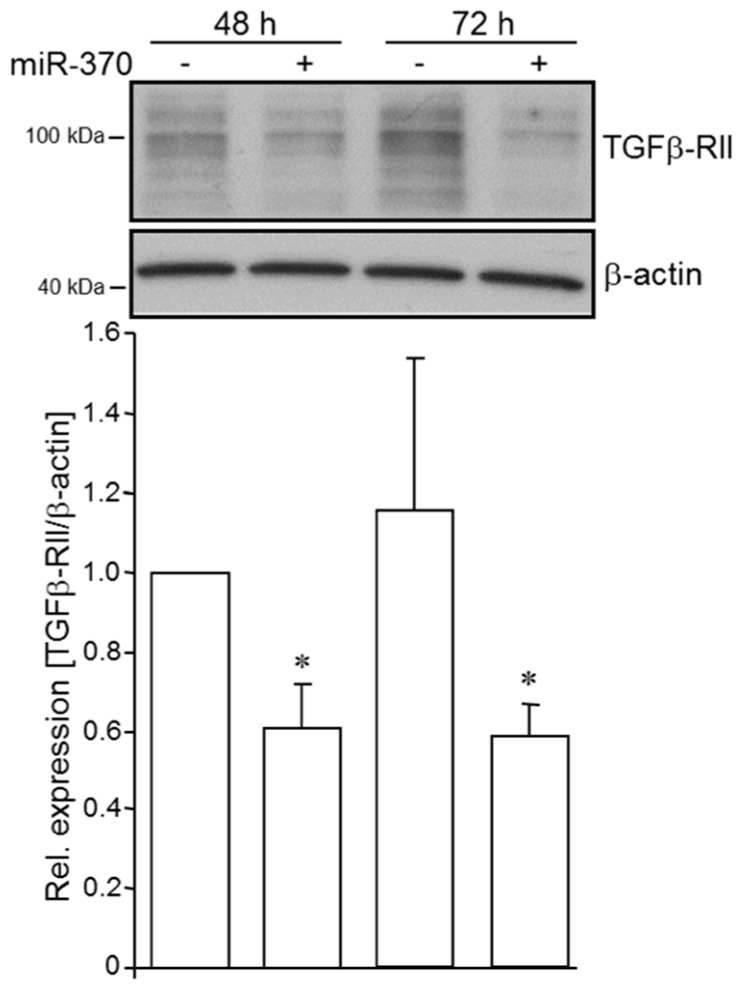
Ectopic expression of miRNA-370-3p in PDAC cells decreases the abundance of TGFβ-RII. Panc1 cells were transfected with 50 nM of an artificial miR-370-3p (miRNA-370-3p mimic) for the indicated periods of time. The levels of TGFβ-RII were analysed by Western blotting in whole cell lysates. Detection of β-actin served as a loading control. The graph underneath the blot shows results from densitometric quantification of band intensities from three independent experiments (mean ± SD, *n* = 3). The asterisks (*) indicate significance (*p* < 0.05) relative to respective untreated control.

Although the regulation of TGFβ-RII by miR-370-3p has been shown in gastric carcinoma [44], data on pancreatic carcinoma are not available so far. To examine if TGFβ-RII is subject to regulation by miR-370-3p in PDAC-derived cells, we transfected Panc1 cells with an artificial miR-370-3p (miR-370-3p mimic) and performed Western blot analysis of TGFβ-RII. As shown in Figure 2, abundance of TGFβ-RII was decreased in miR-370-3p mimic transfected cells relative to control cells at 48 and 72 h after the start of transfection. This indicates that expression of TGFβ-RII protein is inhibited by miR-370-3p.

### 2.3. TRAIL-R1 Knockdown Increases the Abundance of TGFβ-RII

**Figure 3:**
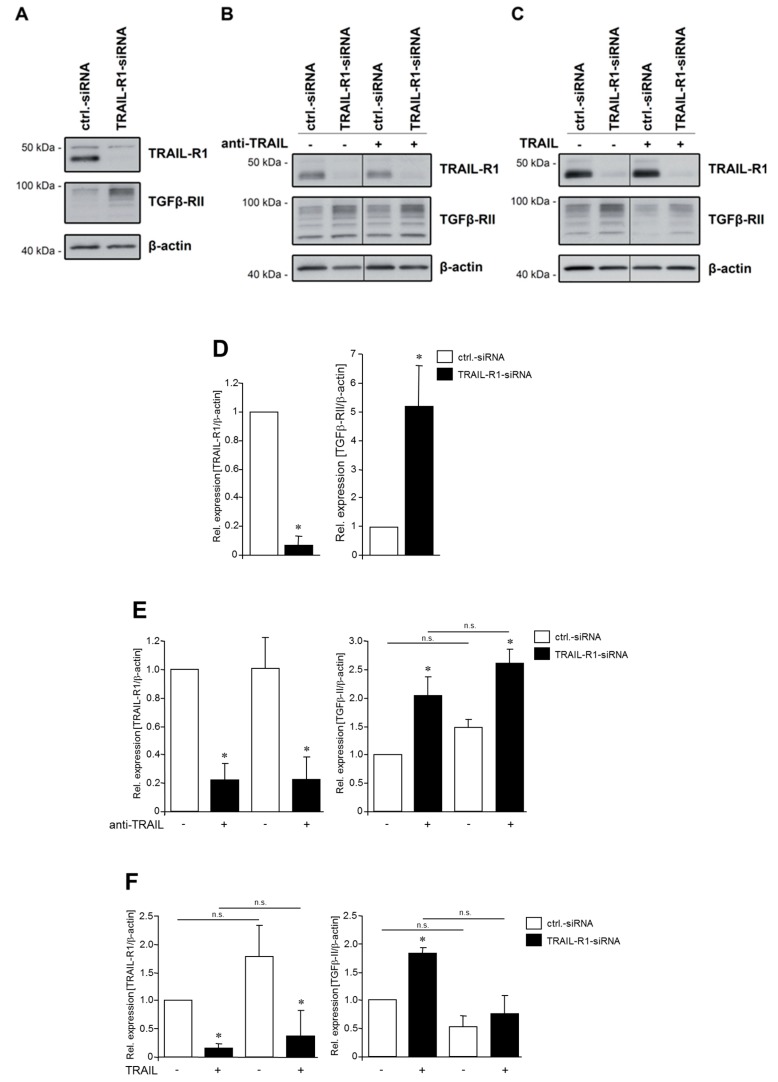
Knockdown of TRAIL-R1 increases the abundance of TGFβ-RII in Panc1 cells. Panc1 cells were transfected with siRNA against TRAIL-R1 or with control siRNA for 72 h without (**A**) or with (**B**) exposure to a neutralizing antibody against TRAIL (anti-TRAIL, 10 µg/mL) or (**C**) recombinant TRAIL (10 ng/mL). The expression of TRAIL-R1 and TGFβ-RII was analysed by Western blotting in whole cell lysates. As control for equal gel loading, levels of β-actin were determined in parallel. The blots shown are representative of three independent experiments yielding very similar results. (**D**) Densitometry-based quantification of the Western blots shown in (**A**). Data were compiled from three independent experiments and represent the mean ± SD (*n* = 3). (**E**) Densitometry-based quantification of the Western blots shown in (**B**). (**F**) Densitometry-based quantification of the Western blots shown in (**C**). The asterisks (*) in (**D**–**F**) indicate significance relative to the ctrl.-siRNA; n.s.: not significant.

Since TGFβ-RII is a target of miR-370 (Figure 2) and knockdown of TRAIL-R1 decreases the cellular levels of miR-370 (Figure 1), we hypothesized that TRAIL-R1 might impact the levels of TGFβ-RII in PDAC cells. To validate this hypothesis, we downregulated the expression of TRAIL-R1 in two PDAC cell lines and analysed the levels of TGFβ-RII by Western blot. As demonstrated in Figure 3A, inhibition of TRAIL-R1 expression via siRNA in Panc1 cells was associated with considerably increased levels of TGFβ-RII. Similar results were obtained with Colo357 cells, which were either transiently transfected with the same siRNA sequences or cells stably transduced with a short-hairpin-RNA (shRNA, sequence different from that of the siRNA) against TRAIL-R1 (Figure S3). This confirms the presence of a functional axis of TRAIL-R1, miR-370 and TGFβ-RII.

To examine a possible ligand dependency of this novel TRAIL-R1 function, Panc1 cells were either stimulated with TRAIL or incubated with anti-TRAIL (Figure 3B). Interestingly, the abundance of TGFβ-RII remained unchanged after incubation with TRAIL or anti-TRAIL. We thus conclude that TRAIL-R1 functions independently of its ligand TRAIL in the regulation of TGFβ-RII.

### 2.4. Knockdown of TRAIL-R1 Enhances Activation of Smad and Non-Smad Pathways after TGFβ Stimulation

**Figure 4:**
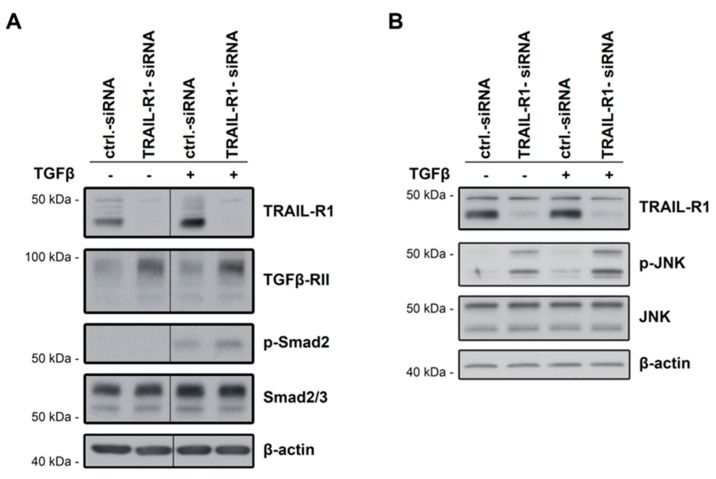

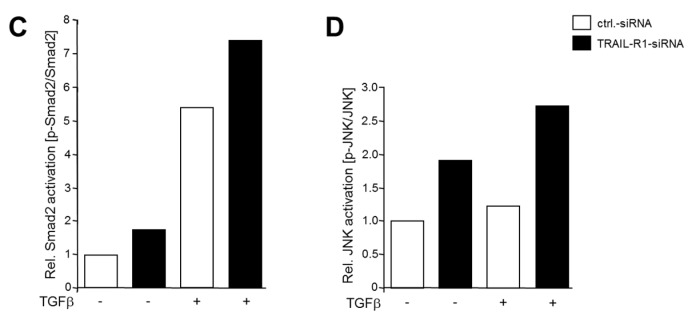
Knockdown of TRAIL-R1 might increase the activation of Smad2 and JNK. Panc1 cells have been transfected with control siRNA (ctrl.-siRNA) or siRNA against TRAIL-R1 (TRAIL-R1-siRNA) for 72 h prior to treatment with TGFβ1 (0.2 ng/mL) for 50 min. Western blot analysis of (**A**) p-Smad2C and (**B**) p-JNK. Detection of β-actin served as control for equal loading. In (**A**) in the Smad2/3 panel, the upper band represents Smad2. The blots in (**A**,**B**) are representative of three independent experiments all yielding, albeit to a varying extent, an induction of p-Smad2C and p-JNK levels, respectively, in TRAIL-R1-siRNA over ctrl.-siRNA transfected cells. (**C**) Densitometry-based quantification of the p-Smad2 bands shown in (**A**). (**D**) Densitometry-based quantification of the p-JNK bands shown in (**B**).

As TGFβ-RII is indispensable for TGFβ signalling, we addressed the question whether an increase in abundance of this protein after TRAIL-R1 knockdown was associated with enhanced TGFβ signalling activity. This was studied by measuring C-terminal phosphorylation of Smad2 (p-Smad2C), a marker for activation of the canonical TGFβ/Smad signalling pathway as well as by assessing the phosphorylation state of JNK1/2 as indicators of non-Smad signalling. We depleted Panc1 cells of TRAIL-R1 by siRNA transfection and 48 h later treated the cells with TGFβ1. As shown in Figure 3, knockdown of TRAIL-R1 led to a strong increase in the levels of TGFβ-RII (Figure 4A, lane 2 vs. 1 and lane 4 vs. 3) and this increase was not affected by treatment with TGFβ1 (Figure 4A, lane 4 vs. 2). Whereas non-stimulated transfectants failed to exhibit detectable levels of p-Smad2C, stimulation with TGFβ1 increased the abundance of p-Smad2C and this increase tended to be stronger in TRAIL-R1 knockdown cells compared with control cells (Figure 4A, lane 4 vs. 3, statistical significance tightly missed in a series of three independent experiments). Neither TGFβ1 stimulation nor depletion of TRAIL-R1 resulted in alterations of the non-phosphorylated forms of Smad2 and Smad3 (Figure 4A). These results suggest the possibility that TRAIL-R1 can inhibit Smad activation which would be consistent with inhibition of TGFβ-RII, as this receptor is crucial for Smad activation by TGFβ1.

We also analysed the non-phosphorylated and phosphorylated forms—the ratio of which reflects the activation state—of the mitogen-activated protein kinase JNK1/2. Depletion of TRAIL-R1 in Panc1 cells was associated with a tendency towards increased levels of p-JNK1/2 when compared to the irrelevant control siRNA (Figure 4B, lane 2 vs. 1 and lane 4 vs. 3, again statistical significance was tightly missed in three independent experiments). These results suggest that TRAIL-R1 might not only inhibit Smad but also non-Smad, for example, JNK signalling secondary to inhibition of TGFβ-RII.

### 2.5. MiR-370-3p Reduces TGFβ-induced Expression of PAI-1 and SLUG

**Figure 5:**
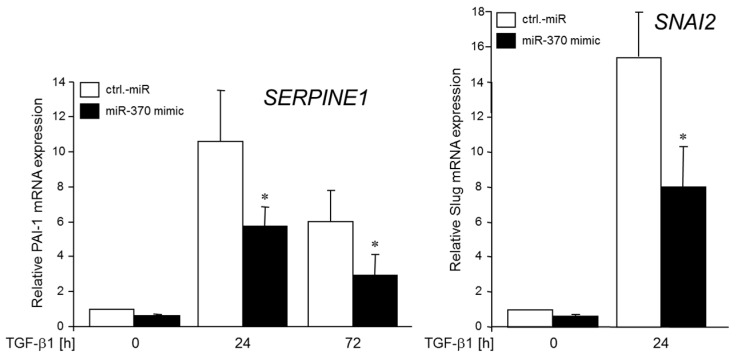
Transfection with a miR-370-3p mimic decreases TGFβ1-induced expression of PAI-1 and SLUG. Panc1 cells were transiently transfected with 50 nM of control (ctrl) miR or a miR-370-3p mimic and 48 h later remained unstimulated (0) or were stimulated with TGFβ1 (5 ng/mL) for 24 or 72 h (PAI-1), or 24 h (SLUG). Cells were examined for PAI-1- and SLUG- expression by qPCR. Data represent the mean ± SD of three independent experiments (*n* = 3). The asterisks (*) indicate significance relative to control (*p* < 0.05).

Above, we have shown that miR-370 controls the expression of TGFβ-RII in a negative fashion. To examine whether alterations in miR-370 levels affect the expression of established TGFβ target genes, we transfected Panc1 cells with the miR-370-3p mimic, or control miR and subsequently treated the cells with rec. TGFβ1 for 24 or 72 h. Interestingly, we noted a lower sensitivity of *SERPINE1* (encoding plasminogen activator-inhibitor 1, PAI-1) and *SNAI2* (encoding SNAIL2/SLUG) to TGFβ1 stimulation as measured by qPCR (Figure 5).

### 2.6. MiR-370 Reduces TGFβ-induced Growth Inhibition

**Figure 6:**
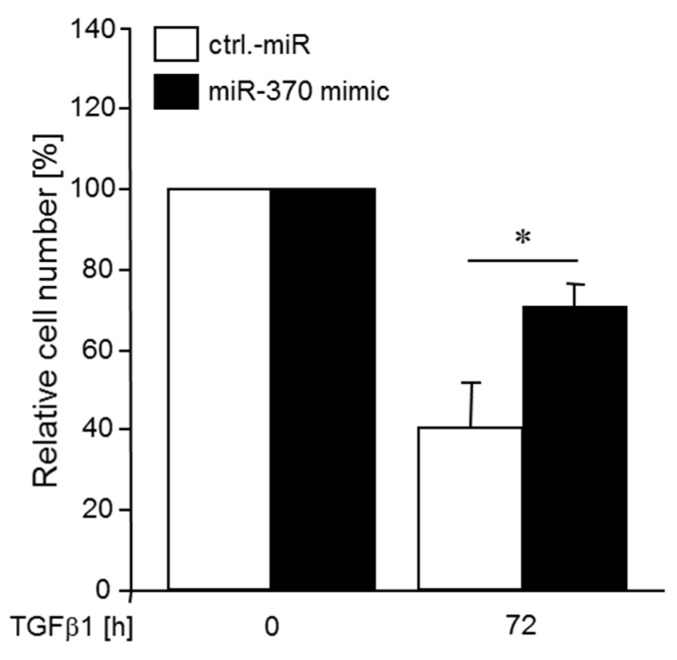
Transfection with a miR-370-3p mimic decreases TGFβ1-induced growth inhibition. Panc1 cells were transiently transfected twice on two consecutive days with 50 nM of control (ctrl) miR or a miR-370-3p mimic and 24 h after the second round of transfection remained unstimulated (0) or were stimulated with TGFβ1 (5 ng/mL) for 72 h. Following TGFβ1 stimulation, cells were detached and viable cells counted. Data represent the mean ± SD of three independent experiments (*n* = 3). The number of non-TGFβ1-treated control cells were set arbitrarily at 100%. The asterisk (*) indicates significance (*p* < 0.05).

In order to test whether miR-370 can also affect more complex cellular responses to TGFβ, for example, growth inhibition, we again transfected Panc1 cells with the miR-370-3p mimic, or control miR and subsequently treated the cells with rec. TGFβ1 for 72 h. Intriguingly, the percentage of viable cells in TGFβ1-treated cultures relative to non-treated control cultures was higher in cultures that had been transfected with the miR-370-3p relative to ctrl.-miR transfected cells (Figure 6). These data clearly show that miR-370-3p by downregulating TGFβ-RII not only suppresses the TGFβ response of individual genes but also impacts growth arrest, a hallmark feature of TGFβ’s tumour suppressor function.

### 2.7. Knockdown of TRAIL-R1 Enhances TGFβ-Induced PAI-1 Expression

**Figure 7:**
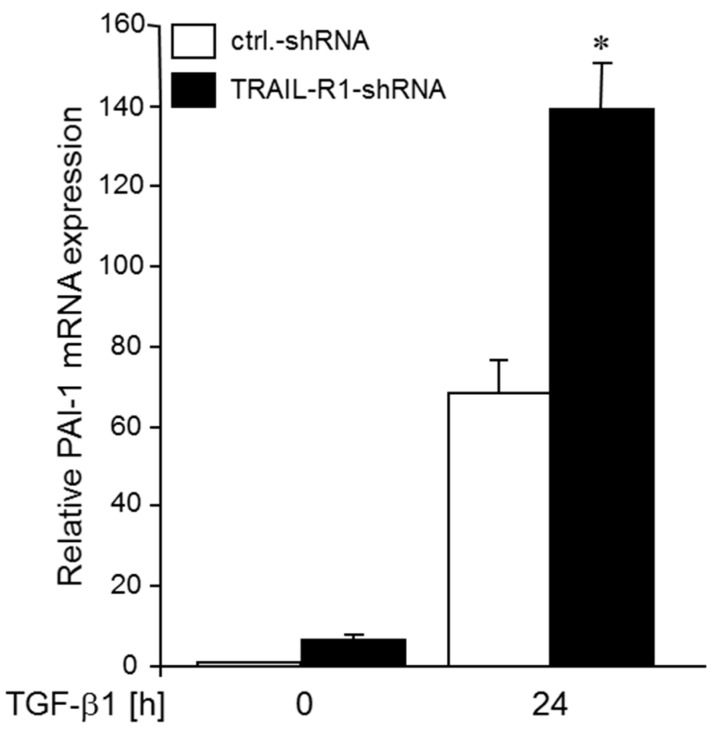
Knockdown of TRAIL-R1 in Colo357 cells enhances TGFβ1-mediated induction of PAI-1 expression. Colo357 cells stably transduced with a TRAIL-R1 shRNA, or control (ctrl.) shRNA remained unstimulated (0) or were stimulated with TGFβ1 (5 ng/mL) for 24 h. Cells were assayed for PAI-1 mRNA expression by qPCR. Data represent the mean ± SD of three independent experiments (*n* = 3). The asterisk (*) indicates significance relative to control (*p* < 0.05).

Above, we have shown that ectopic expression of miR-370-3p decreased TGFβ1-induced PAI-1 expression (see Figure 5). Since miR-370, in turn, is positively controlled by TRAIL-R1 (see Figure 1), we hypothesized that knockdown of TRAIL-R1 increases the sensitivity of *SERPINE1* to TGFβ1 stimulation as a result of a concomitant decrease in miR-370 levels. To this end, Colo357 cells stably expressing a TRAIL-R1 shRNA (Colo357-shTRAIL-R1) exhibited higher levels of PAI-1 after TGFβ1 stimulation (Figure 7).

### 2.8. Ectopic Expression of a miR-370-3p Mimic Partially Reverses the TRAIL-R1 shRNA-induced TGFβ Hyperstimulation of PAI-1 Expression

**Figure 8:**
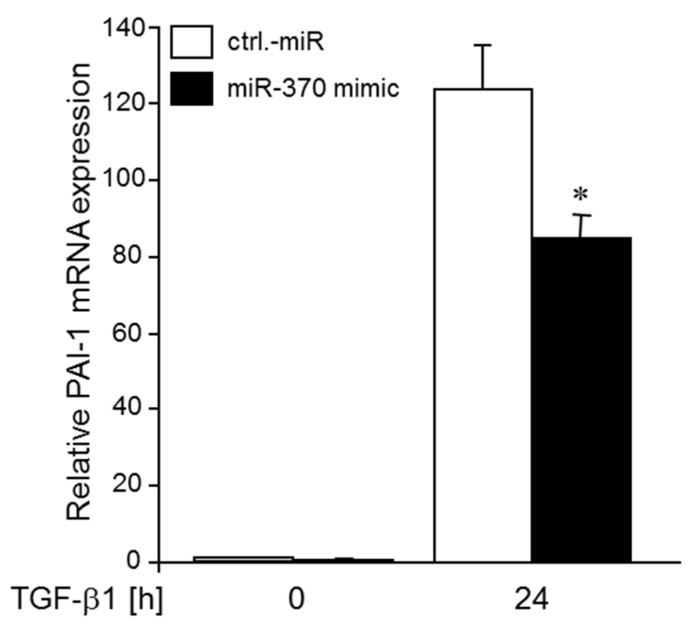
Ectopic expression of a miR-370-3p mimic partially rescues Colo357 cells from the TRAIL-R1 shRNA-induced TGFβ1 hyperstimulation of PAI-1 expression. Colo357 cells with stable expression of a TRAIL-R1 shRNA were transiently transfected with 50 nM of control (ctrl.) miR or a miR-370-3p mimic and 48 h later stimulated with TGFβ1 (5 ng/mL) for 24 h. Data represent the mean±SD of three independent experiments (*n* = 3). The asterisk (*) indicates significance relative to control (*p* < 0.05).

If TRAIL-R1 inhibits TGFβ1-dependent PAI-1 expression through miR-370-3p-mediated down-regulation of TGFβ-RII, then the enhancement of the TGFβ1 effect on PAI-1 expression following TRAIL-R1 depletion (see Figure 7) should be rescued by transfection of a miR-370-3p mimic. To this end, transfection of Colo357-shTRAIL-R1 cells with miR-370-3p mimic partially reversed the TGFβ1-induced hyperstimulation of PAI-1 expression when compared to a control miR (Figure 8). Together with the results shown in Figure 7, these data confirm the functional link between miR-370-3p, TRAIL-R1 and sensitivity to TGFβ being largely determined by expression of TGFβ-RII.

## 3. Discussion

The role of miR-370-3p in malignant cells seems to vary depending on the tumour entity. Whereas some studies found a tumour-promoting function, others conclude that miR-370-3p works as a tumour suppressor [45,46,47]. According to the latter scenario, levels of miR-370 in gastrointestinal stromal tumours, bladder carcinomas and neuroblastomas were reported to be low [48,49,50]. As shown by Xu and co-workers, amounts of mature miR-370-3p declined during the development from pre-malignant liver lesions to end-stage HCC [47]. Furthermore, these authors demonstrated that inhibition of miR-370 resulted in a larger tumour mass, increased invasive potential and higher tumour stage. Moreover, in tissue samples from patients, low levels of miR-370 correlated with reduced survival [47].

Previous studies indicated that miR-370-3p negatively controls expression of TGFβ-RII in gastric carcinoma cells [45], however, equivalent data for PDAC cells are not available. High levels of miR-370-3p would therefore be expected to be associated with lower levels of TGFβ-RII and presumably attenuated TGFβ signalling activity and target gene expression. In agreement with this assumption we found that transfection of cells with a miR-370-3p mimic reduced the sensitivity of *SERPINE1* and *SNAI2* to TGFβ1 stimulation (see Figure 5) and partially relieved TGFβ1-induced growth arrest (see Figure 6). 

Little is known on the mechanisms that control mature miR-370 expression. In addition to their well-known function as membrane-bound death-inducing receptors, TRAIL-R1 and -2 were also found to localize in the nuclei of malignant cells [19]. Recently, it was uncovered that TRAIL-R2, by associating with the nuclear proteins p68, NF45 and hnRNPA1, negatively regulated the maturation of miR let-7, while expression of let-7 target proteins were elevated resulting in a more malignant phenotype [20]. Here, we report that TRAIL-R1, too, engages in modulating miR expression since knockdown of this receptor was accompanied by downregulation of miR-370-3p representing a novel mechanism of positive regulation of miR-370 by TRAIL-R1. The observation that TRAIL-R1 but not treatment of the cells with exogenous TRAIL, impact the expression of miR-370-3p argues in favour of a ligand independent function of TRAIL-R1. Interestingly, however, antibody-mediated deprivation of endogenous TRAIL slightly reduced miR-370-3p levels, the reason of which is not clear at present.

Given the negative association of miR-370 with TGFβ-RII expression on the one hand and its positive association with TRAIL-R1 expression on the other hand, we hypothesized that TRAIL-R1 via modulation of miR-370 should also affect the expression of TGFβ-RII in PDAC cells. Regulation of TGFβ-RII via TRAIL/TRAIL-R system has not been described so far. As predicted, we observed that TRAIL-R1 negatively regulates abundance of TGFβ-RII. We assume that the extra TGFβ-RII protein that appears after TRAIL-R1 knockdown, is cell surface-associated as the TGFβ receptor complex which is central to the regulation of TGFβ signalling is localized at the plasma membrane. This is in line with the changes seen in the sensitivity of cells to TGFβ stimulation with respect to gene expression and growth inhibition. We also analysed whether stimulation of PDAC cells with TRAIL would affect expression of TGFβ-RII, however, TRAIL-R1 appears to function independently of its ligand TRAIL here.

To gain further insight into a possible functional relevance of TGFβ-RII upregulation upon TRAIL-R1 knockdown for the activation of TGFβ signalling, analysis of the canonical Smad signalling pathway revealed a tendency towards an activation after stimulation with TGFβ1. The same tendency was noted for non-Smad TGFβ signalling pathways, for example, JNK1/2, which appeared to be activated after TRAIL-R1 knockdown under both non-stimulated and TGF β1-stimulated conditions. An attempt to quantify the increase in p-Smad2C and p-JNK levels in TRAIL-R1-depleted cells from three experiments, however, yielded p-values slightly greater than 0.05. We are currently performing additional experiments to increase the probability that differences become statistically significant. In addition, we are monitoring activation of Smad3 to further support inhibition of canonical Smad signalling by TRAIL-R1. A possible increase in JNK activation following TRAIL-R1 knockdown independent of TGFβ1 stimulation (see Figure 4, lane 1 vs. 2) may be due to the binding of endogenous TRAIL to TRAIL-R2. This explanation is supported by the fact that TRAIL-R2 but not TRAIL-R1 signals through JNK activation [51]. Finally, we show that TRAIL-R1 knockdown-induced upregulation of TGFβ-RII led to a dramatic increase in the expression of the (Smad-dependent) TGFβ target gene *SERPINE1* in Colo357 cells (see Figure 7). Intriguingly, this effect could be partially blocked by transient transfection of the cells with the miR-370-3p mimic (see Figure 8).

Recently, we have uncovered an interdependence of TGFβ and TRAIL-induced signalling in PDAC cells. We found that TGFβ1 decreased the levels of TRAIL-R1 and negatively impacted TRAIL-R1-mediated apoptosis and non-apoptotic signalling [52]. Together, our previous and current data create interesting scenarios depending on the level of expression of TGFβ. Since PDAC cells show high expression levels of TRAIL-R1 [19,20], which positively controls miR-370 expression, we propose that miR-370 is abundant in early-stage PDAC when TGFβ is low. In this miR-370-rich environment, mRNA transcribed from *TGFBR2* will be degraded and, hence, TGFβ signalling and TGFβ-driven tumourigenesis inhibited. In line with this assumption, high levels of cytoplasmic TRAIL-R1 expression in tumour cells have been shown to correlate with better prognosis of patients with PDAC [53] and other tumour entities (reviewed in Ref. 19).

In contrast, in later stages of PDAC when TGFβ1 levels are high in tumours, this scenario will change dramatically. TGFβ1 is then expected—via inhibiting TRAIL-R1 [52]—to downregulate also miR-370. Interestingly, the fraction of pancreatic cancer samples with positive membrane staining for TRAIL-R1 was found to be lower than that of cells from surrounding non-tumour tissues [54]. Likewise, miR-370 expression, too, is underexpressed in both pancreatic cancer precursor lesions [37] and cancer cells [55] compared to normal healthy tissue and cells, respectively. A more direct link between TGFβ and miR-370-3p recently came from an ovarian cancer cell model [56]. Here, TGFβ treatment downregulated miR-370-3p and a miR-370-3p knockdown or miR-370-3p overexpression promoted or suppressed, respectively, TGFβ-induced epithelial-mesenchymal transition [56] the latter process of which is considered a crucial event in TGFβ’s protumourigenic function. Downregulation of miR-370 may relieve TGFβ-RII expression from inhibition and eventually further stimulate TGFβ expression [57] resulting in hyperactivation of TGFβ signalling. This vicious cycle of TGFβ signalling [57] may be responsible for driving aggressive behaviour, invasive potential and metastasis in late-stage PDAC and other gastrointestinal stroma-rich tumours.

## 4. Conclusions

In summary, we present here a novel mechanism of TRAIL-R1-dependent, miR-370-mediated regulation of the expression of TGFβ-RII that finally resulted in corresponding alterations in TGFβ-dependent target gene expression and growth inhibition. Many miRs were found to be deregulated in PDAC cells correlating with worsened patient outcome, chemoresistance and invasive behaviour [58]. Although selective modulation of miRs is still not trivial [59], pharmacological intervention to decrease or increase distinct miRs to improve therapy outcomes in patients with pancreatic carcinomas is nevertheless a promising strategy. Eventually, miR-370 may emerge as a promising candidate among other TGFβ signalling inhibitors that are currently evaluated in anticancer therapy [60,61,62].

## 5. Materials and Methods

### 5.1. Culture Conditions, Cell Stimulation and Counting

The human pancreatic cancer cell lines Panc1 and Colo357 were cultured in RPMI 1640 media supplemented with 10% FCS, 1 mM GlutaMAX and 1 mM sodium pyruvate (Life Technologies, Darmstadt, Germany). For selection of stably retrovirally transduced Colo357 cells the antibiotic puromycin was added to the culture media (2.5 µg/mL). Cells were grown for 24 h in six-well-plates before transfection and/or stimulation. Cell-stimulating agents used were TGFβ1 (ReliaTech, Wolfenbüttel, Germany), recombinant human TRAIL (10 ng/mL; PeproTech, Hamburg, Germany) and neutralizing TRAIL antibody (anti-TRAIL) (10 µg/mL; R&D Systems). For cell counting, cells were detached by trypsinization and viable cells (as determined by trypan blue exclusion) counted in a Neubauer chamber.

### 5.2. Transfection of siRNA, shRNA and miR-370

1.7 × 10^5^ cells were seeded in six-well-plates the day before transfection. Control siRNA and siRNA against TRAIL-R1 were used in a concentration of 2 nM, both of them purchased from GE Healthcare (ON-TARGETplus^®^ SMARTpool non-targeting pool D-001810-10, L-008090-00; GE Healthcare, Freiburg, Germany). To obtain cells with a stable knockdown of TRAIL-R1, Colo357 cells were transduced with GIPZ lentiviral shRNAmir vectors for TRAIL-R1 or with a non-silencing control (Open Biosystems; CloneID: V3LHS_383714). MirVana^®^ miR mimic hsa-miR-370-3p and the respective control were purchased from Ambion, Life Technologies, Darmstadt, Germany. DharmaFECT^®^1 (Thermo Scientific, Schwerte, Germany) or Lipofectamin 2000 (Life Technologies/ThermoFisher Scientific, Darmstadt, Germany served as transfection reagents and were used according to the manufacturers’ instructions. Forty-eight h after transfection, cells were stimulated with the aforementioned factors and then subjected to protein extraction or RNA isolation.

### 5.3. Western Blot Analysis

For extraction of proteins, cells were lysed in RIPA buffer including a protease- (cOmplete Mini, Roche Diagnostics) and a phosphatase inhibitor (PhosSTOP, Roche Diagnostics). Performance of Western blot analysis was described before [63]. Primary antibodies were purchased from Cell Signalling, Frankfurt, Germany (anti-JNK, anti-phospho-JNK, anti-phospho-Smad2); Merck Millipore, Darmstadt, Germany (anti-TRAIL-R1); Santa Cruz Biotechnology, Dallas, TX, USA (anti-TGFβ-RII, anti-Smad2/3); Sigma-Aldrich, St. Louis, MO, USA (β-actin).

### 5.4. RNA Isolation, Reverse Transcription and Polymerase Chain Reaction

RNA was extracted from cells by a mirVana^TM^ miR kit (Ambion). Quantitative real time PCR of the complementary DNA (TaqMan, Applied Biosystems, Foster City, CA, USA) was performed on a 7900HT Fast Real-Time PCR system (Applied Biosystems) using TaqMan qPCR Master Mix (ThermoFisher Scientific Dharmacon, Epsom, UK) and MIR370 TaqMan MicroRNA Assay (ThermoFisher Scientific, Cat.-# 4427975) pri-miR-370 Assay (ThermoFisher Scientific, Hs03303493_pri), RNU6B Assay (ThermoFisher Scientific) or GAPDH Assay (ThermoFisher Scientific, Hs99999905_m1). Other primers used were: PAI-1 (forward: cttcttcaggctgttccggagc; reverse: gggtcagggttccatcacttgg), Slug (forward: atattcggacccacacattacct; reverse: gcaaatgctctgttgcagtga) and TATA box-binding protein (TBP, forward: gctggcccatagtgatcttt; reverse: cttcacacgccaagaaacag). The latter two housekeeping genes were amplified as controls. For quantification, the delta-delta C_t_ method was employed to show expression levels of the genes-of-interest after normalization with RNU6B (for mature miR-370), GAPDH (for pri-miR-370) or TBP (for PAI-1 and SLUG).

### 5.5. Statistical Analysis

All data were normally distributed and analysed using two-tailed student’s *t*-test. A *p* value < 0.05 was considered statistically significant. Data are shown as mean ± standard deviation (SD). Statistical analysis was performed using Sigma Plot 12.5 software (Systat Software, Erkrath, Germany).

## Supplementary Materials

The following are available online at http://www.mdpi.com/2072-6694/10/11/399/s1. Figure S1: TRAIL-R1 regulates the expression of miRs; Figure S2: TRAIL-R2 knockdown has no effect on miR-370-3p expression in Panc1 cells; Figure S3: TRAIL-R1 knockdown elevates protein levels of TGFβ-RII in Colo357 cells.
