# Supplementary material for: Downregulation of TRAIL-Receptor 1 Increases TGFβ Type II Receptor Expression and TGFβ Signalling Via MicroRNA-370-3p in Pancreatic Cancer Cells
Source: Cancers (Basel). 2018 Oct 25;10(11):399. doi: 10.3390/cancers10110399 (PMC6267290; doi:10.3390/cancers10110399)
Supplement: Supplementary file 1 [file cancers-10-00399-s001.zip › cancers-369800-SI.pdf]

Supplementary materials: Downregulation of TRAIL-receptor 1 increases TGFβ type II receptor expression and TGFβ signaling via microRNA-370 in pancreatic cancer cells

David I. Radke, Qi Ling, Robert Häsler, Gökhan Alp, Hendrik Ungefroren and Anna Trauzold

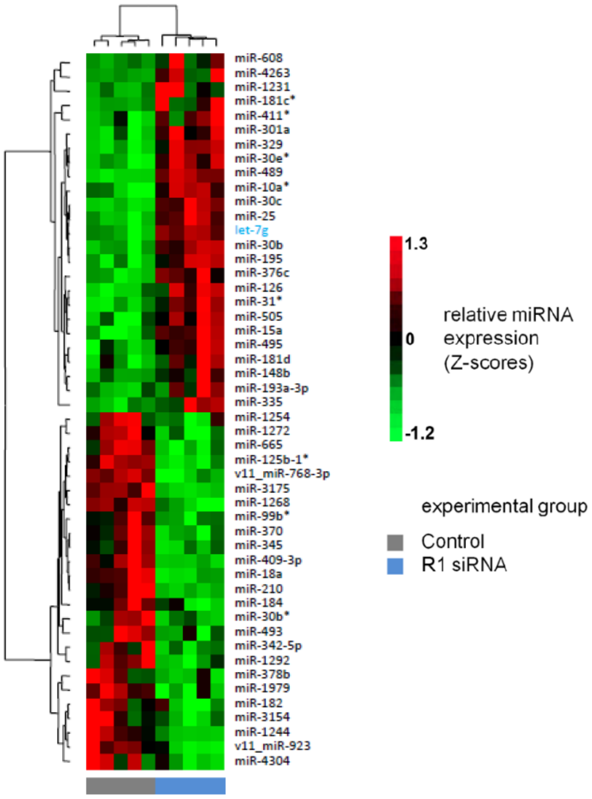

Figure S1. TRAIL-R1 regulates the expression of miRs. Panc1 cells were treated for 40 h with siRNA against TRAIL-R1 (R1 siRNA) or with control siRNA (Control). RNA was analyzed for expression of miRs by Affymetrix miRNA 2.0 Galaxy arrays. For each siRNA, the top 50 (25 most upregulated and 25 most downregulated) miRs are displayed. Each miR showed a significant regulation ($p < 0.05$). For better readability, relative miR expression values were normalized using Z-score normalization. Relative expression values are color-coded according to the red-green scale. Row dendrograms represent regulatory similarities between individual miRs (based on correlation) while column dendrograms represent sample similarities. Each experimental group consists of 5 biological replicates. This miRNA array detects both the 3p and 5p forms of miR-370. *: indicates that the predominantly expressed mature miRNA sequence is from the opposite arm of the predicted precursor.

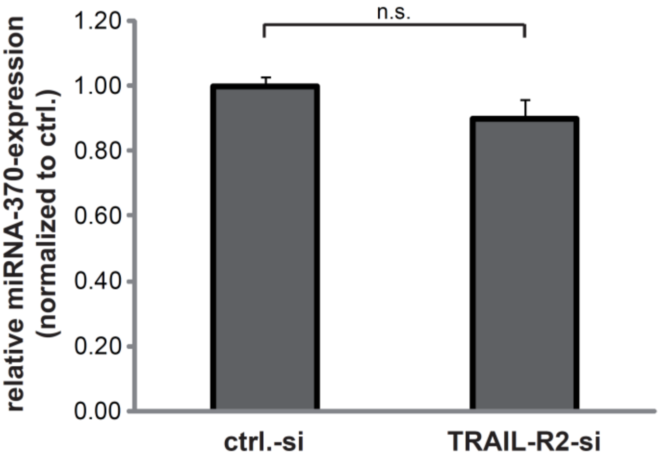

Figure S2. TRAIL-R2 knockdown has no effect on miR-370-3p expression in Panc1 cells. Quantitative PCR analysis detecting changes in the levels of mature miR-370 in Panc1 cells transfected for 40 h with a control-siRNA (ctrl.-si) or siRNA against TRAIL-R2 (TRAIL-R2-si). Levels of mature miR-370 were quantified by qPCR. Shown are means±SD of five biological replicates, each analyzed in technical duplicates; n.s., not significant.

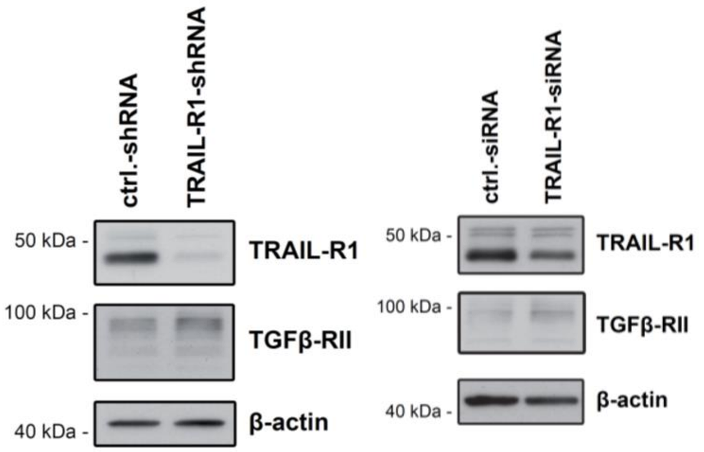

Figure S3. (**A**) TRAIL-R1 knockdown elevates protein levels of TGFβ-RII in Colo357 cells. Western blot analysis of whole cell lysates derived from Colo357 cells stably transfected with a TRAIL-R1-shRNA was performed with antibodies to TRAIL-R1, TGFβ-RII, and β-actin as an internal control. The blot is representative of three independent experiments yielding very similar results. (**B**) TRAIL-R1 knockdown elevates protein levels of TGFβ-RII in Colo357 cells. Western blot analysis of whole cell lysates derived from Colo357 cells transiently transfected with a TRAIL-R1-siRNA was performed with antibodies to TRAIL-R1, TGFβ-RII, and β-actin as an internal control. The blot is representative of three independent experiments yielding very similar results.

Material and Methods:

MicroRNA array

Panc1 cells were treated for 40 h with siRNA (On-Targetplus® smart pool siRNA; Dharmacon) against TRAIL-R1 or with control siRNA in experimental setups with 5 biological replicates per group. To obtain genome-wide miRNA profiles for cells in which TRAIL-R1 was downregulated, total RNA was extracted using the miRVANA kit (AMBION) and quality controlled with an Agilent Bioanalyzer (Agilent). Further processing on Affymetrix miRNA 2.0 Galaxy arrays was performed according to the

manufacturers' guidelines (Affymetrix). Data were processed using Affymetrix Command Console (Affymetrix) and normalized using RMA (Bioconductor.org). Differential expression was determined by calculating the fold-changes based on the ratios of the medians per group, while statistical differences were determined using the Mann-Whitney U-test.
